# The Extended Term of Training

**Published:** 1920-06-05

**Authors:** 


### THE MATRONS' AND SISTERS' DEPARTMENT.

### THE EXTENDED TERM OF TRAINING.

The position of the leading nursing authorities who for many years opposed the movement for State Registration has been gravely misunderstood. The grounds of their opposition were weighty. Their caution has been amply justified by events. Their judgment in favour of the unfettered development of British training-schools for nurses is now triumphantly vindicated. What has been gained by delay?

Looking back over the past twenty-five years or so which have elapsed since State registration became a war-cry in the nursing world, it can be realised that during this time a sound, workable system of training nurses has been evolved in this country, and that nursing progress has been vitally facilitated by the absence of a hard-and-fast system imposed on the nursing world from without. There has been time to test rival systems. Even twenty years ago a large proportion of training-schools were giving a two-year course. Very few indeed had started the fourth year. Are we justified in believing that the universal acceptance of the three years' course, the increase in the number of schools with a four years' course, and the inception of the fifth or scholarship year might never have blossomed into fruition had State registration been forcibly imposed upon reluctant hospital managers?

Experience has shown that in cases where State registration has preceded instead of following the natural growth and development of professional zeal, it has had one of two disastrous effects: It has irretrievably retarded development by setting too low a standard, or it has created widespread discontent and resistance by setting a standard higher than nurses or the public were prepared for.

The effect of State registration on many American institutions illustrates the extent to which registration may operate to obstruct. Once a State has adopted a two-year standard it is extremely difficult to secure its alteration. Many vested interests oppose a higher standard, and, though the alteration of a law is not so costly or laborious a process in the States as in this country, it is by no means easy to procure the necessary unanimity to effect a tightening of the rein. The same holds good in Canada. It stands to reason that nursing reformers in this country would have been seriously handicapped in preaching the need for the third year of hospital training had the State once begun to hallmark the two-year trained nurse.

But, it will be argued, no one in this country ever advocated a State system which would recognise two years' training. It is highly probable that the promoters of the agitation might have striven for a three years' qualification. It is by no means probable that they would have realised their ideal. Had there been no opposition to registration twenty-five years ago, had a Government department framed the regulations with due regard to existing conditions and possibilities, no higher standard than two years could have been imposed. The result would have been to wreck English nursing as a profession.

On the other hand, the mischievous effects of prematurely forcing up the standard have been witnessed in many directions. When the American Red Cross Society attempted to limit admission to the Army Nursing Service to registered nurses it was found that the proportion of nurses who had ignored registration was so overwhelming that it was impracticable to maintain this distinction. It has never been realised by the advocates for State registration at any price that before nurses will offer themselves for voluntary registration there must be a long preparatory period during which a sound public opinion must be developed in doctors, patients, and nurses themselves in favour of a thorough training. A system of registration which attracts merely those nurses anxious to qualify for a particular kind of work is a dead failure and a costly one. Unless it be quasi-universal and embrace the whole profession we are better without it.

In this country, as a direct consequence, we believe, of delay in setting up registration, the three-year course is already accepted by every single training-school whose size would enable its certificate to be accepted by the Board. In a large proportion of schools a fourth year is not only obligatory but eagerly desired, as may be seen by the competition among nurses for this extra year of institution work instead of appointment to the private staff. Besides this we have the amazing fact that members of the College are subscribing to give their *confrères* an opportunity of enjoying yet a fifth year of study in preparation for their career. Can registration in any country show the equal of this?

